# PKM2 and HIF-1α regulation in prostate cancer cell lines

**DOI:** 10.1371/journal.pone.0203745

**Published:** 2018-09-14

**Authors:** Diya Hasan, Elisabetta Gamen, Nafez Abu Tarboush, Yazan Ismail, Oleg Pak, Belal Azab

**Affiliations:** 1 Al-Balqa Applied University, Zarqa College, Department of Allied Medical Sciences, Zarqa, Jordan; 2 Independent Researcher, Giessen, Germany; 3 The University of Jordan, School of Medicine, Department of Biochemistry and Physiology, Amman, Jordan; 4 Excellence Cluster Cardio-Pulmonary System (ECCPS), German Lung Center (DZL), Universities of Giessen and Marburg Lung Center (UGMLC), Justus Liebig University, Giessen, Germany; University of Texas MD Anderson Cancer Center, UNITED STATES

## Abstract

Prostate cancer (PCA) is one of the most common cancer types in men, with cancer progression being linked to hypoxia and the induction of hypoxia-inducible factor (HIF).We investigated the expression of pyruvate kinase M2 (PKM2), its regulation by HIF isoforms 1α and 2α, and its role in HIF stabilization. We additionally examined cell survival in the prostate cancer cell lines PC3 and LNCaP under severe hypoxic (0.1% O_2_) and normoxic (20% O_2_) conditions. qRT-PCR showed higher up-regulation of PKM2 mRNA expression in LNCaP cells than in PC3 cells, while western blotting showed that PKM2 protein levels were up-regulated only in LNCaP cells. Inhibition of HIF-1α and HIF-2α by small interfering RNA (si-RNA) demonstrated HIF-1α dependent up-regulation of PKM2 at the mRNA and protein levels in LNCaP cells. PKM2 inhibition by si-RNA significantly decreased hypoxia-response element (HRE) activation in a gene reporter assay and down-regulated HIF-1α target vascular endothelial growth factor (VEGF) mRNA expression in PC3 cells, whereas HIF-1α protein levels were not significantly reduced. Additionally, PKM2 inhibition significantly reduced clonogenic survival in both cell lines in a colony formation assay. Prolyl hydroxylase 3 (PHD3) mRNA expression was up-regulated in both cell lines. It has been shown that PKM2 expression is regulated by HIF-1α and that PKM2 favors HIF-1α transactivation under mild (1% O_2_) but not severe (0.1% O_2_) hypoxic conditions, and some of our findings are consistent with these previous results. However, this mechanism was not fully observed in our studied cell lines, as PKM2 regulation and HIF-1α stabilization at the transactivation level occurred under severe hypoxic conditions. This discrepancy suggests that tumor tissue origin and cell type influence this model. Our findings expand the current knowledge of the mechanisms of PCA regulation, and would be important in developing novel therapeutic strategies.

## Introduction

Prostate cancer (PCA) is one of the most common cancer types in men, with an annual estimated 1.1 million cases and 307,000 deaths worldwide [1]. Low oxygen concentration, or hypoxia, has been shown to correlate with resistance to chemotherapy *in vitro* and radiotherapy *in vivo*, as well as to promote malignancy progression [2]. The degree of hypoxia ranges within tumors between 0.3% and 4.2% O2, with a median level of <2% depending on the tissue of origin [3]. In addition, there is a notable diversity of oxygen levels within individual tumors [4], depending on the efficiency and proximity of local blood vessels [5]. This reflects the fact that tumor cells exhibit differing hypoxia-inducible factor 1α (HIF-1α) expression and regulation under mild 1% O2 and severe 0.1% O2 hypoxic conditions [6]. Invasive prostate adenocarcinoma shows higher HIF-1α expression than does the normal epithelium, stromal cells, and benign prostatic hyperplasia [7]. Overexpression of HIF-1α in PCA is associated with shorter relapse time in patients receiving surgery or radio therapy in addition to chemo/ castration resistance and metastasis [8].

**Fig 1:**
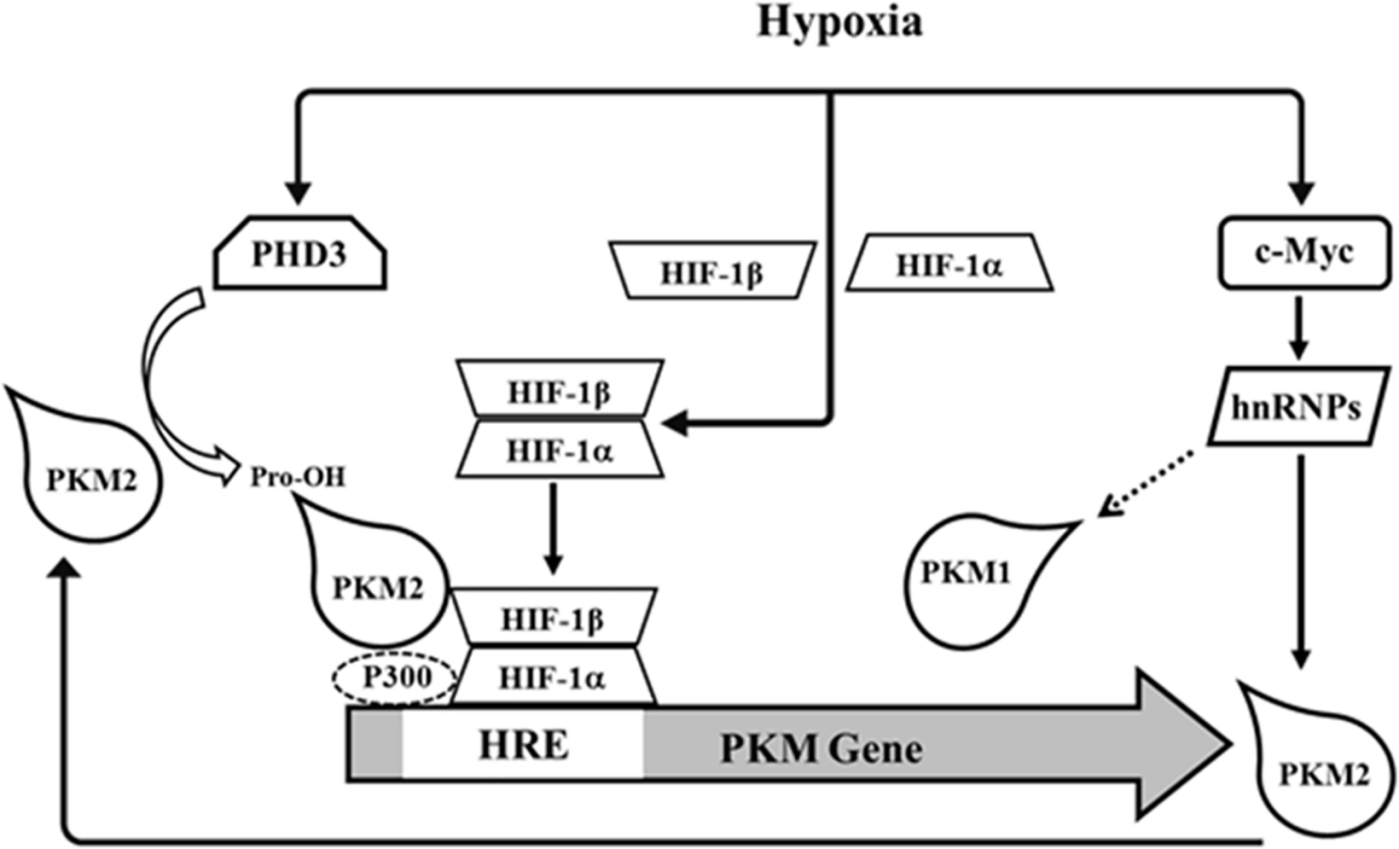
Schematic representation of regulation of PKM2 and its interaction with HIF-1α.

HIF-1α regulates pyruvate kinase M2 (PKM2) expression by binding to the hypoxia-response elements (HREs) located within the first intron of the PKM2 gene [9] (Fig 1), thus regulating cell metabolism. PKM2 catalyzes the conversion of phosphoenolpyruvate into pyruvate, which is last step of the glycolytic pathway [10]. PKM2 expression has been associated with esophageal squamous cell carcinoma (ESCC) chemoresistance [11], while its knockdown in non-small cell lung cancer (NSCLC) increased the radiosensitivity of resistant cell lines [12]. Moreover, up-regulation and specific modification of PKM2 has also been associated with PCA progression [13].

Activation of HIF-1α occurs due to protein stabilization [14] (Fig 1) by inhibition of the prolyl hydroxylase family (PHD1-3), which hydroxylates specific prolyl residues in the oxygen-dependent degradation (ODD) domain of HIF-1α to enhance its degradation [15, 16]. PHD3 also hydroxylates specific proline residues on PKM2 in mild (1% O2) but not severe (0.1% O2) hypoxic conditions, favoring transactivation of HIF-1α [9] (Fig 1). Additionally, PKM2 increases the binding of HIF-1α and the recruitment of the coactivator p300 to HREs on HIF-1α target genes in a positive feedback loop [9] (Fig 1).

Surgery and radiation therapy are traditional approaches for clinically localized cancers, whereas androgen deprivation associated with chemotherapy is usually used in cases of metastatic disease [17]. Hormone and radiation therapy have good success rates in patients with intermediate-risk factors, however the disease can still recur in those with more advanced tumor burdens [18]. Thus, therapies making the tumor more sensitive to radiation can be beneficial. A better understanding of PCA biological environmental conditions; such as hypoxia and HIF-1α, which play a central role in PCA oncogenesis; growth; and metastasis; is important to decreasing the mortality rate of the disease [19]. Thus, this study was an in-depth investigation of PKM2 and HIF-1α regulation mechanisms and their cross-talk in PCA cell lines under severe hypoxic conditions, rather than the conditions investigated previously [9].

Hypoxia activates HIF-1α dimerization with HIF-1β to form HIF-1α [20], which induces PKM2 at the transcriptional level mediated by its HRE elements [9]. C-myc is relevant to PKM2 since it regulates critical hnRNP proteins [21] that affect differential splicing of PKM1 and PKM2 [22], favoring the latter [23]. Overall, these pathways determine the absolute and relative levels of PKM1 and PKM2. The interaction between PKM2 and HIF-1α involving PHD3 hydroxylation of PKM2 at proline residues is displayed. This modification results in HIF-1α transactivation by interaction with the transcription factor complex [9].

## Materials and methods

### Cell culture and hypoxic incubation

The human prostate carcinoma cell lines PC3 and LNCaP were used in this study as in previous HIF-1α expression studies [7, 24–26]. PC3 and LNCaP cell lines were obtained from the American Type Culture Collection (Manassas, VA, USA) and cultured following the manufacturer’s recommendations. Cells were exposed to hypoxia (HOX) in a chamber equilibrated with a water-saturated gas v/v mixture of 0.1% O_2_, 5% CO_2_, and the rest nitrogen at 37°C (Innova CO-48; New Brunswick Scientific, Edison, NJ, USA). Control cells were maintained under normoxic conditions (NOX) in water-saturated room air, supplemented with 5% (v/v) CO_2_ at 37°C.

### RNA interference by synthetic si-RNA

PC3 and LNCaP cells were transfected with small interfering RNA (si-RNA) targeting PKM2 and HIF-1α or HIF-2α and control siRNA (sequences shown in S1 Table) synthetized by Biomers (Ulm, Germany). Transient transfection of siRNA was performed with Lipofectamine 2000 (Invitrogen, Carlsbad, CA, USA) according to the manufacturer’s protocols. Cells were subcultured to 70% confluence in DMEM-F12 supplemented with 10% FBS. Transfection of siRNAs was performed in Opti-MEM medium for 5 h, followed by culturing in DMEM-F12 supplemented with 10% FBS (Thermo Fisher Scientific, Waltham, MA, USA).

### RNA isolation, reverse transcription and real-time PCR

Total RNA was extracted from the hypoxia-exposed and si-RNA treated cells, and equal amounts (2 μg) were subsequently transcribed into cDNA using M-MuLV reverse transcriptase (Thermo Fisher Scientific). The cDNA was used as a template in real-time PCR performed with the ABI Prism 7300 detection system (Applied Biosystems, Foster City, CA, USA) with SYBR green as a fluorescent dye (Invitrogen). Human-specific primers for PKM2, HIF-1α, HIF-2α, VEGF, and PHD3 (sequences shown in S2 Table) were designed using sequence information from NCBI and were purchased from Biomers (Biomers). Expression was analysed by the ΔCt method. The Ct values of the target genes were normalized to that of the porphobilinogen deaminase (PBGD) gene (endogenous control).

### Western blot analysis and quantification

Total protein was extracted by the peqGOLD TriFast procedure (Peqlab Biotechnology GmbH, Erlangen, Germany). Proteins were separated on 10% polyacrylamide gels and transferred to polyvinylidene fluoride (PVDF) membranes (EMD Millipore, Billerica, MA, USA). After blocking, the membranes were probed with one of the following antibodies: anti-HIF-1α (BD Biosciences, Franklin Lakes, NJ, USA), anti-HIF-2α (LifeSpan BioSciences, Inc., Seattle, WA, USA), anti-PKM2 (Cell Signaling Technologies, Danvers, MA, USA), anti-PKM2 (Y105; Cell Signaling Technologies), and anti-β-actin (Abcam, Cambridge, United Kingdom). All primary antibodies were diluted 1:1000. Then membranes were incubated with secondary antibodies conjugated with horseradish peroxidase (HRP). Antibody complexes were visualized by enhanced chemiluminescence using an ECL kit (GE Healthcare, Little Chalfont, United Kingdom). An image reader (FluorchemTM IS-8900, Alpha Innotech, San Leandro, CA, USA) was used to visualize and quantify western blot bands. Expression was quantified using band intensity values (in arbitrary units), which were normalized to that of β-actin.

### HRE reporter gene assay

The pGL3-TK plasmid (Promega, Madison, Wisconsin, USA) with a thymidine kinase minimal promoter (TK-MP) and five repeats of HRE from the phosphoglycerate kinase (PGK) gene were used to construct the HRE-reporter plasmid [27]. The results were normalized against *Renilla* luciferase, which was co-transfected to correct for variations in transfection efficiency. PC3 and LNCaP cells were grown to 80% confluence in 48-well plates and co-transfected with HRE-reporter plasmid, si-control, and si-PKM2 using Lipofectamine 2000, and then incubated under normoxic or hypoxic conditions for 24 h. The firefly luciferase (FLuc) activity in transfected cells was measured using the luciferase assay system (Promega) and a spectrofluorometer (FL-600 BioTek Instruments, Winooski, VT, USA). Fluorescence values are reported as relative light units (RLUs).

### Colony formation assays

PC3 and LNCaP cells were seeded at a density of 1 × 10^4^ cells per 100 mm dish and cultured in 0.35% soft agar in DMEM-F12 plus 10% FBS (Thermo Fisher Scientific) at 37°C for 48 h. Afterwards, cells were transfected with si-PKM2 or si-control once every 3 days a total of two times. After the first transfection, cells were incubated for two weeks in normoxia or hypoxia. Finally, cells were stained with crystal violet, rinsed in water, and air-dried. All visible colonies were counted. The surviving fraction was calculated as follows: (mean colony counts) / (cells inoculated) = plating efficiency (PE), where PE is a measure of the number of colonies originating from single cells.

### Statistical analysis

Statistical analysis was performed using GraphPad Prism 5.03 (GraphPad Software, Inc., La Jolla, CA, USA). All data are represented as means ± standard error (mean ± SEM). Student’s t-test and one-way analysis of variance (ANOVA) were used to determine the levels of statistical differences between two and multiple groups, respectively.

## Results

### PKM2 expression under normoxic and hypoxic conditions

**Fig 2:**
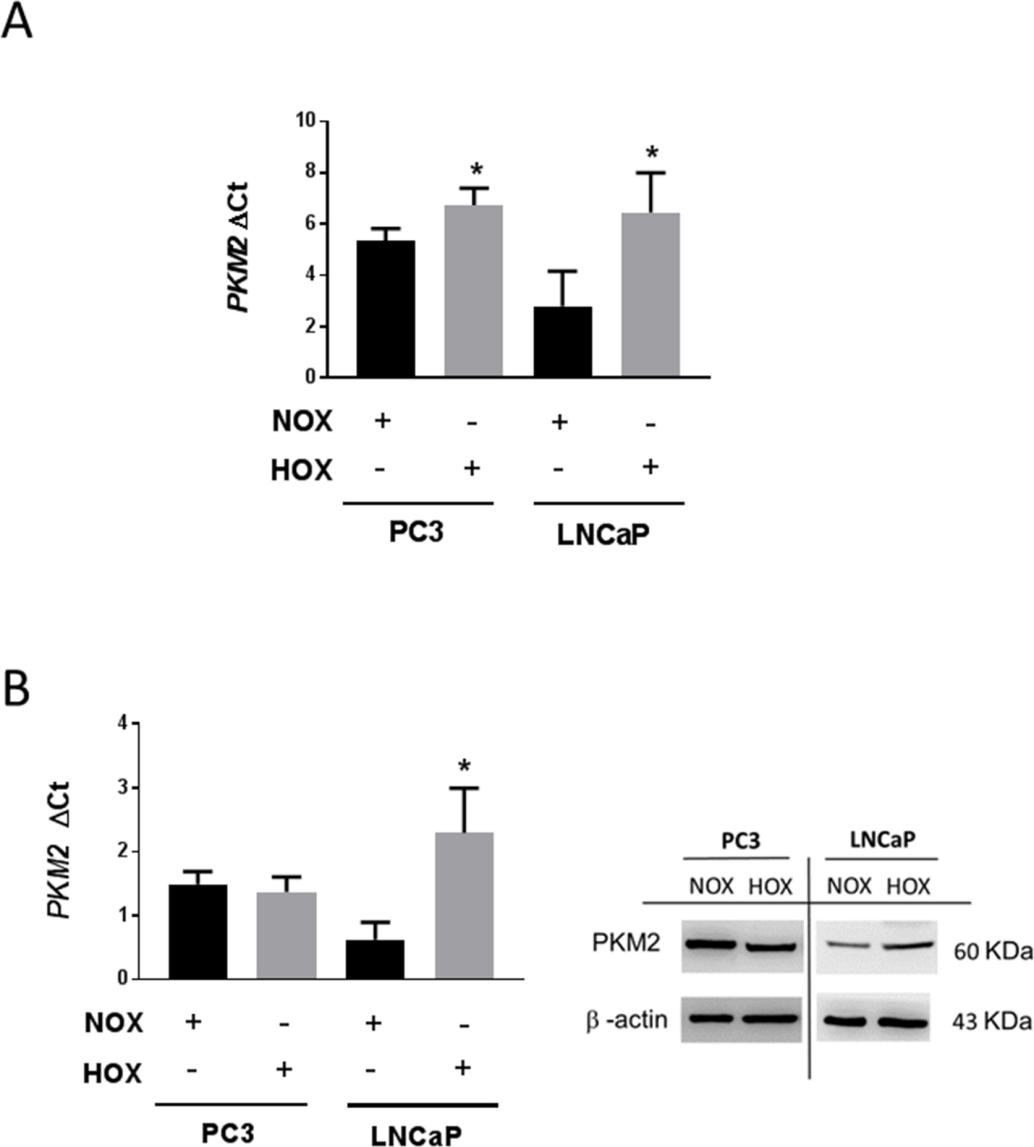
PKM2 expression in normoxia and hypoxia in prostate cancer cell lines. PC3 and LNCaP cells were cultured for 24 h in normoxia 20% O2 or hypoxia 0.1% O2. (A) PKM2 mRNA expression detection and quantification by qRT-PCR (n = 3, Mean ± SEM, * *P* < 0.05 vs NOX). (B) Densitometric analysis of western blot for PKM2 normalized to β-actin. (n = 3, Mean ± SEM, * *P* < 0.05).

PKM2 mRNA was expressed at a higher level in PC3 cells than in LNCaP cells under normoxic conditions (Fig 2A). Severe hypoxia significantly increased PKM2 mRNA expression in both cell lines, with a more pronounced increment in LNCaP cells (Fig 2A). However, PKM2 protein levels showed significant up-regulation in LNCaP cells, but not in PC3 cells (Fig 2B) in severe hypoxic as compared to normoxic conditions.

### Inhibition of HIF-1α and HIF-2α by si-RNA and its effect on PKM2

**Fig 3:**
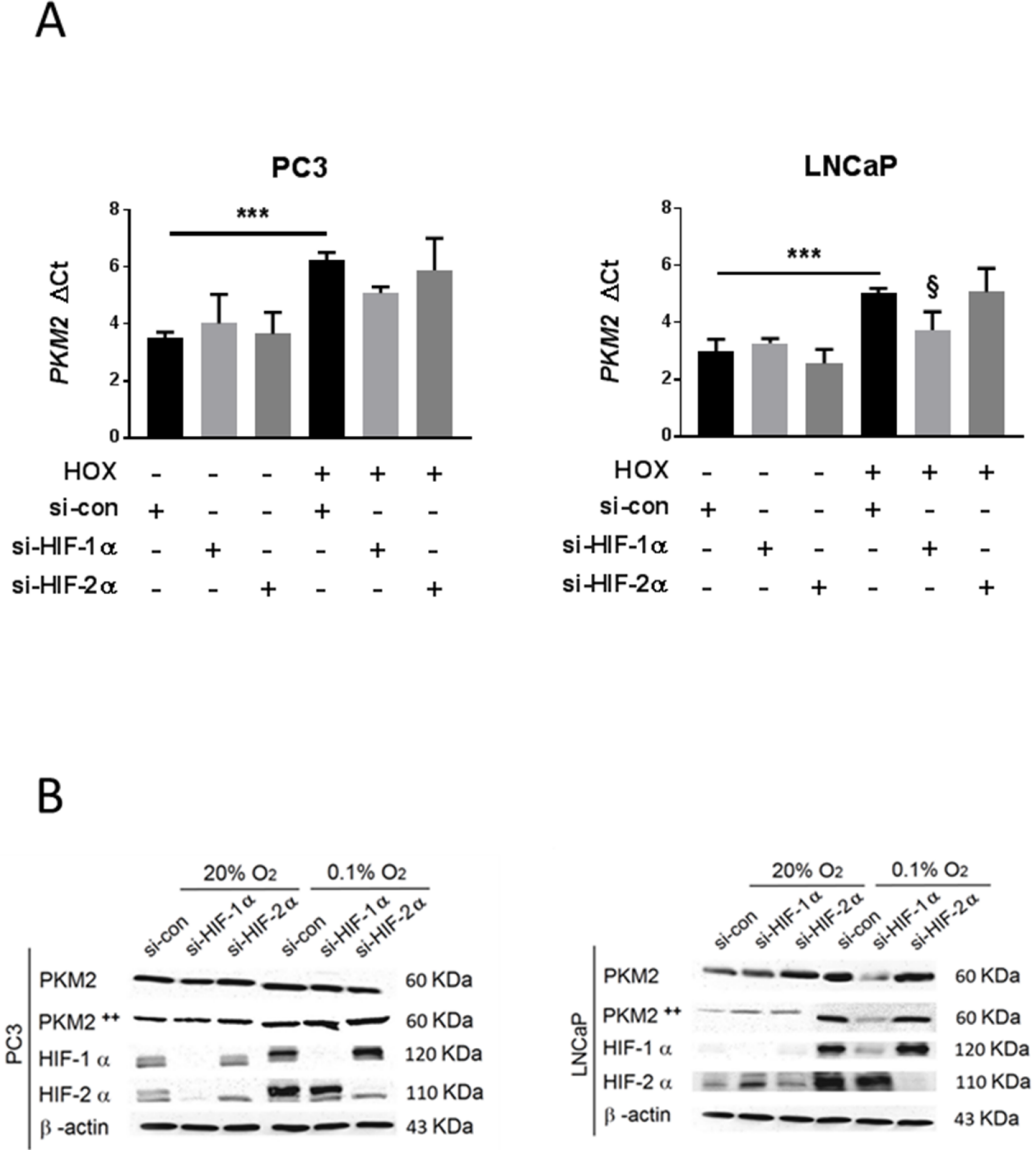
Effects of HIF-1α and HIF-2α inhibition by si-RNA on PKM2 expression. PC3 and LNCaP cells were transfected and cultured for 24 h in normoxia 20% O2 or hypoxia 0.1% O2. (A) PKM2 mRNA expression detection and quantification by qRT-PCR after treatment of cells with si-HIF-1α or si-HIF-2α (n = 4, Mean ± SEM, *** *P* < 0.001, ^**§**^ *P* < 0.05 vs si-con Hox). (B) Western blot analysis of PKM2 and PKM2 ++ (Tyr105) levels after si-HIF-1α or si-HIF-2α transfection and validation of the suppressive effects of si-HIF-1α and si-HIF-2α on HIF-1α and HIF-2α respectively.

Silencing of HIF-1α and HIF-2α was validated by qRT-PCR and western blotting (S1A Fig and Fig 3B respectively). Since PC3 cells displayed high HIF-1α expression even in normoxia, we further confirmed the efficiency of the silencing by HRE luciferase assay (S1B Fig). qRT-PCR analysis of mRNA extracts from PC3 cells demonstrated no effect on PKM2 mRNA expression from si-HIF-1α or si-HIF-2α as compared to that from si-control in hypoxia. However, LNCaP cells showed a significant down-regulation of PKM2 mRNA expression after si-HIF-1α but not si-HIF-2α transfection as compared to expression after si-control transfection in hypoxia (Fig 3A). No significant changes were observed in normoxia in either cell line. Consistently, western blot analysis demonstrated no changes in PKM2 or phosphor-PKM2 (Tyr105, modified PKM2 with higher activity) protein levels in PC3 cells after si-HIF-1α or si-HIF-2α transfection as compared to levels after si-control transfection in severe hypoxia and normoxia (Fig 3B). However, PKM2 and phospho PKM2 (Tyr105) protein levels were down-regulated in LNCaP cells after si-HIF-1α but not si-HIF-2α transfection as compared to levels after si-control transfection in hypoxia (Fig 3B).

### PKM2 inhibition by si-RNA and its effect on HIF-1α

**Fig 4:**
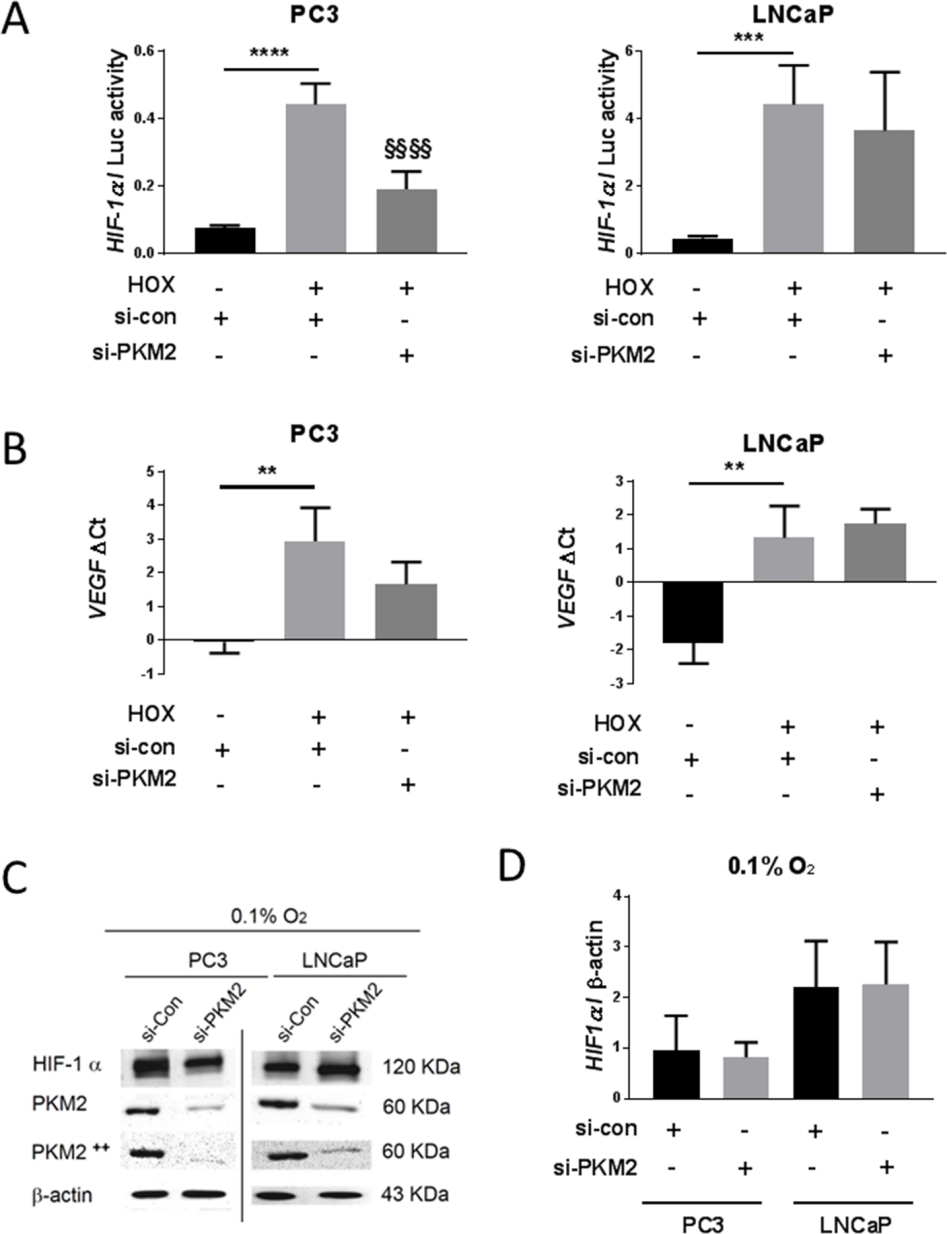
Effect of PKM2 inhibition by si-RNA on HIF-1α. PC3 and LNCaP cells were transfected and cultured for 24 h in normoxia 20% O2 or hypoxia 0.1% O2. (A) HRE reporter gene assay of transfected cells with si-PKM2 (n = 6, Mean ± SEM, *** *P* < 0.001, **** *P* < 0.0001, ^**§§§§**^ *P* < 0.0001 vs si-con Hox). (B) VEGF mRNA expression detection and quantification by qRT-PCR after treatment of cells with si-PKM2 (n = 3, Mean ± SEM, ** *P* < 0.01 vs si-con HoX). (C) Western blot analysis of HIF-1α protein levels after si-PKM2 transfection and validation of the suppressive effects of si-PKM2 on PKM2 and PKM2++ (Tyr105) protein levels. (D) Densitometric analysis of HIF-1α western-blot normalized to β-actin after treatment of cells with si-PKM2.

Since silencing HIF-2α did not affect PKM2 expression, we focused on HIF-1α regulation. After confirming the efficiency of PKM2 silencing by qRT-PCR and western blotting (S2 Fig and Fig 4B, respectively), HIF-1α protein levels were analyzed. Densitometric analysis showed no significant difference between the effects of si-PKM2 and si-control transfection on HIF-1α protein levels in PC3 or LNCaP cells in hypoxia (Fig 4C and 4D). Further, HRE luciferase activity was measured. We observed that si-PKM2 significantly down-regulated HRE activity in PC3, but not in LNCaP cells, as compared to si-control under hypoxic conditions (Fig 4A). To further confirm these results, we analyzed mRNA expression of VEGF, a well-known HIF-1α target. Consistently, VEGF mRNA expression was down-regulated by si-PKM2 compared to si-control in PC3 cells but not in LNCaP cells under hypoxic conditions (Fig 4B).

### Expression of PHD3 under normoxic and hypoxic conditions

**Fig 5:**
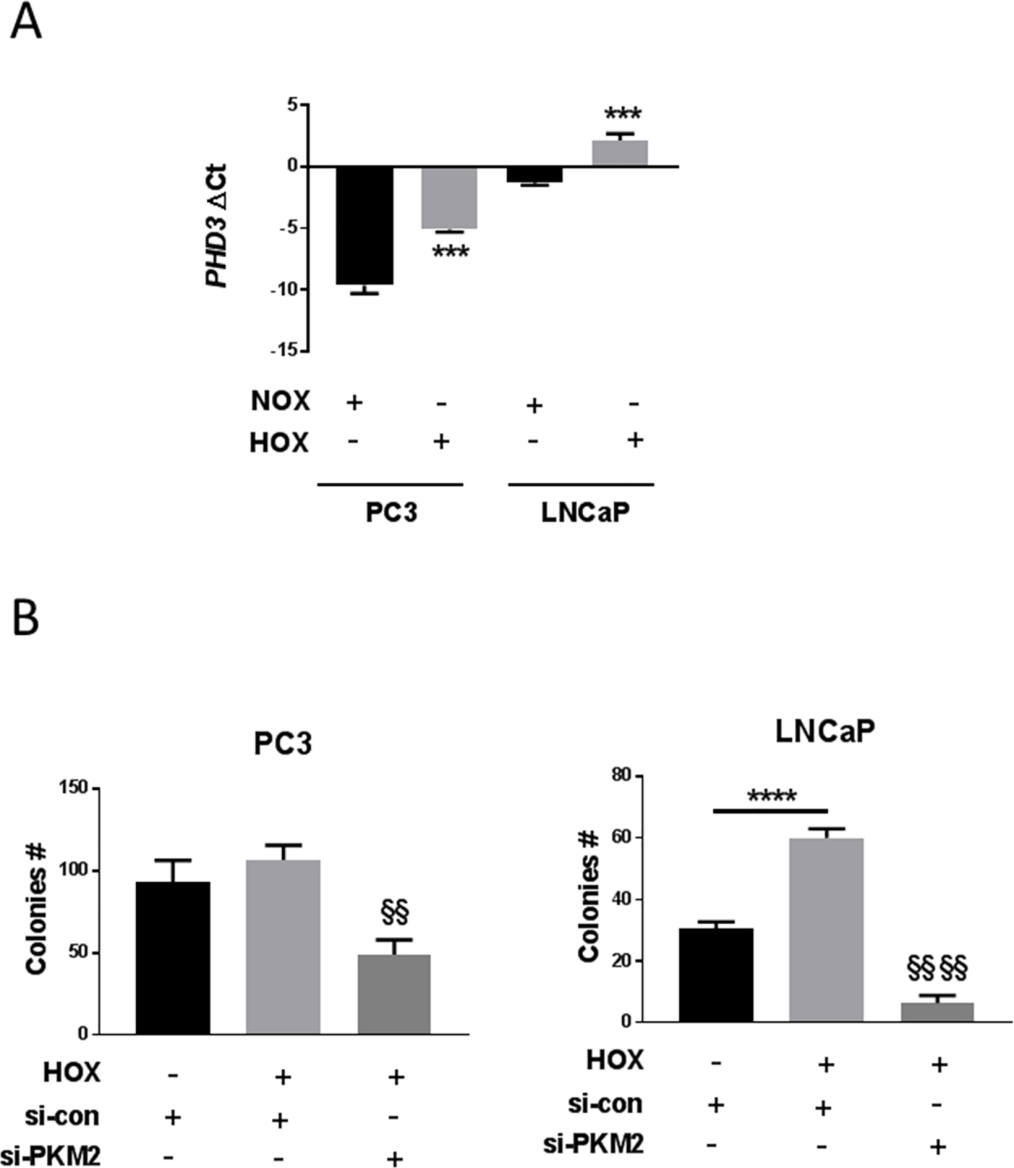
PHD3 expression and colony survival in prostate cancer cell lines. PC3 and LNCaP cells were transfected and cultured for 24 h in normoxia 20% O2 or hypoxia 0.1% O2. (A) PHD3 mRNA expression detection and quantification by qRT-PCR (n = 3, Mean ± SEM, *** *P* < 0.001 vs NOX). (B) Colony survival assay in transfected cells with si-PKM2, (n = 3, Mean ± SEM, ** *P* < 0.01, **** *P* < 0.0001, ^**§§§§**^ *P* < 0.0001 vs si-con Hox).

qRT-PCR analysis of mRNA extracts from PC3 and LNCaP cells showed a significant increase in PHD3 mRNA expression in severe hypoxia as compared to that in normoxia in both cell lines, with a more pronounced increase in PC3 cells (Fig 5A).

### Effect of PKM2 inhibition on cell proliferation

To investigate the role of PKM2 on cell proliferation in severe hypoxic conditions, we performed a colony formation assay. PKM2 silencing affected the growth of PC3 and LNCaP cells. There was an 80–90% decrease in LNCaP colony formation and a 50% decrease in PC3 colony formation when the cells were transfected with siRNA directed against PKM2 as compared to si-control in hypoxia (Fig 5B).

## Discussion

Oxygen levels within tumors are very heterogeneous, ranging from mild to severe hypoxia [28] or anoxia [29] (0.1% O2). Tumor hypoxia is associated with increased radioresistance and metastatic potential, resulting in poor prognosis. Previous studies have demonstrated the presence of HIF-1α dependent molecular events triggered by severe hypoxia. HIF-1α regulates PKM2 gene expression, and PKM2 in turn interacts with the HIF-1α subunit and stimulates its transactivation in a feedback loop [30]. In the present study, we explored the effects of severe hypoxia on the crosstalk between HIF-1α and PKM2 in the context of prostate cancer.

PKM2 basal mRNA expression was higher in PC3 than in LNCaP cells under normoxic conditions. Its expression was significantly induced by severe hypoxia in both cell lines (Fig 2A). Hypoxic regulation of PKM2 mRNA has already been described in hepatoblastoma cells (HepG2) [31] and mouse embryonic fibroblast (MEFs) [9]. This induction was more pronounced in LNCaP than in PC3 cells. It is further well-known that the transcriptional response of hypoxia target genes varies among different human cancer cells [6], accounting for differences observed in PKM2 up regulation in LNCaP and PC3 cell lines. The increment of PKM2 mRNA expression was coupled with increase in the protein levels in LNCaP but not in PC3 cells (Fig 2B), suggesting the activation of different post-transcriptional and/or post-translational mechanisms. Moreover, previous studies have revealed that this correlation between RNA and protein profiles occurs only in one-third of human cell lines [32].

To analyze the role of HIF transcription factors in regulation of PKM2 in prostate cell lines, knockdowns of both, HIF-1α and HIF-2α were performed. Silencing of HIF-1α, but not HIF-2α, significantly down-regulated severe hypoxia-induced PKM2 mRNA (Fig 3A) and protein levels (Fig 3B) in LNCaP but not in PC3 cells. Our results support the idea of PKM2 regulation by HIF-1α in LNCaP cells, as has been suggested previously [9], but not in PC3 cells. These differences between our studied cell lines could be attributed to the higher basal level of HIF-1α we noticed under normoxia in PC3 cells. Several theories were suggested to explain normoxic HIF-1α up-regulation in PCA, including HIF-1α stabilization relying on tumor hypoxia [33], increased HIF-1α mRNA expression [34], gene amplification [35], and single-nucleotide polymorphisms (SNPs) [36]. A previous study revealed that HIF-1α expression under normoxia in PC3 cells is due to HIF-1α gene amplification [35]. Such a cell behavior of HIF-1α expression and regulation mechanism can influence the PKM2/HIF-1α relationship, and the gene expression response to hypoxic stress in a cell-specific manner.

Further, we assume that the high normoxic PKM2 expression (Fig 2A) in PC3 cells is due to HIF-1α normoxic upregulation (Fig 3B). This could also explain why hypoxic stress failed to intensely increase PKM2 mRNA expression and protein levels (Fig 2B). The lack of any effect of silencing HIF-1α on PKM2 expression in PC3 cells is consistent with this (Fig 3B).

Silencing PKM2 did not affect HIF-1α protein levels in PC3 or LNCaP cells (Fig 4D). However, it significantly reduced HIF-1α activity (Fig 4A) and VEGF mRNA expression (Fig 4B) in PC3 but not LNCaP cells in severe hypoxia. HIF-1α expression is linked to increased expression of VEGF [37], and silencing of HIF-1α resulted in decreased expression of VEGF mRNA [38]. These results suggest that HIF-1α activity is controlled at the transactivation level. It has been demonstrated that PKM2 hydroxylation is crucial to HIF-1α transactivation in VHL-null RCC4 renal carcinoma cells at 1% O_2_ but not at 0.1% O_2_ levels [9]. HIF-1α is also regulated by PKM2 via metabolites, including lactate and pyruvate. PKM2 is crucial enzyme to metabolic and glycolytic flux, and its upregulation causes a several-fold increase in glucose consumption and lactate production [39–42].

Hydroxylation of PKM2 by PHD3 in hypoxia is favored by enhanced PHD3 expression, while PHD3 degree of induction varies depending on cell type and pO_2_ [16] [43–45]. PHD3 expression and contribution to malignancy have been detected in several cancers, including pancreatic biliary tumors [46], gastric cancer [47], NSCLC [48], breast cancer [49], and renal cell carcinoma (RCC) [50]. Severe hypoxia induced PHD3 mRNA levels in both PC3 and LNCaP cell lines (Fig 5A). However, PHD enzyme activity is also affected by succinate and α-ketoglutarate [51–53] and small ubiquitin-related modification (SUMOylation) [54].

Differences in tumor oxygenation can explain the discrepancies between our results and previous ones [9]. Oxygenation is normally reported as a median value [3] and varies among tumors depending on the tissue of origin [3]. The median oxygen value of renal cancer is 1.3%O2 [55], cervical cancer 1.2%O2 [4], and prostate cancer 0.3%O2 [56]. A recent study treated LNCaP xenografts with bicalutamide to keep median oxygen below 0.1%O2 for more than 10 days [57]. Androgens can be another factor to explain our results, since PC3 cells are androgen-independent, while LNCaP cells are androgen dependent. Study shows androgens to stimulate HIF-1α expression via an autocrine loop involving EGF/PI3K/AKT in LNCaP cells [58].

Although the mechanism of PKM2 regulation may differ depending on cell type, there is no doubt that it is essential for tumor progression. In the present study, we reported that severe hypoxia promoted cell proliferation in PC3 androgen-independent and LNCaP androgen-dependent prostate cancer cell lines. Consistent with this, a colony survival assay showed that silencing of PKM2 expression significantly decreased colony formation in both PC3 and LNCaP cells under 0.1% O_2_ hypoxia (Fig 5B). This may fit functionally with the observation that PKM2 activity shifts glycolytic metabolites away from energy production towards anabolic processes of cellular compounds required for proliferation [59–61].

Several studies have observed excessive HIF-1α expression in PCA, making it a potential therapeutic target. A diverse variety of HIF-1α inhibitors have been developed and investigated clinically [62]. Proper understanding of the hypoxic stress response in correlation with tissue type is critical to understanding the mechanism of tumor cell adaptation to hypoxia and to the development of efficient therapeutic interventions.

## Conclusions

According to our study, PKM2 expression is regulated by HIF-1α, but HIF-1α expression is not regulated by PKM2 in LNCaP cells. This mechanism of regulation is reversed in PC3 cells, in which PKM2 expression is not regulated by HIF-1α, but HIF-1α expression is regulated by PKM2. Our results put forward cell-specific differences in hypoxic regulation of HIF-1α and PKM2, which might form an important basis for developing specific targeting strategies in future.

## Supporting information

S1 Fig Validation of si-HIF-1α and si-HIF-2α efficiency and knockdown. PC3 and LNCaP cells were transfected and cultured for 24 h in normoxia 20% O2 or hypoxia 0.1% O2. (A) HIF-1α and HIF-2α mRNA expression detection and quantification by qRT-PCR after treatment of cells with si-HIF-1α or si-HIF-2α (n = 4, Mean ± SEM, * *P* < 0.05, ** *P* < 0.01, *** *P* < 0.01 vs si-con Nox or Hox, ANOVA). (B) HRE reporter gene assay of transfected cells with si-PKM2 and si-HIF-1α (n = 6, Mean ± SEM, **** *P* < 0.0001, ^**§§§§**^ *P* < 0.0001 vs si-con Hox).
(TIF)

S2 Fig Validation of si-PKM2 efficiency and knockdown. PC3 and LNCaP cells were transfected and cultured for 24 hr in normoxia 20% O2 or hypoxia 0.1% O2. PKM2 mRNA expression detection and quantification by qRT-PCR after treatment of cells with si-PKM2 (n = 4, Mean ± SEM, *** *P* < 0.001, **** *P* < 0.0001 vs si-con Hox).
(TIF)

S1 Table The sequences of siRNA used to knock down gene targets. (TIF)

S2 Table The sequences of human primers used for real-time PCR. (TIF)
