# Supplementary figures and images for: PKM2 and HIF-1α regulation in prostate cancer cell lines
Source: PLoS One. 2018 Sep 14;13(9):e0203745. doi: 10.1371/journal.pone.0203745 (PMC6138389; doi:10.1371/journal.pone.0203745)

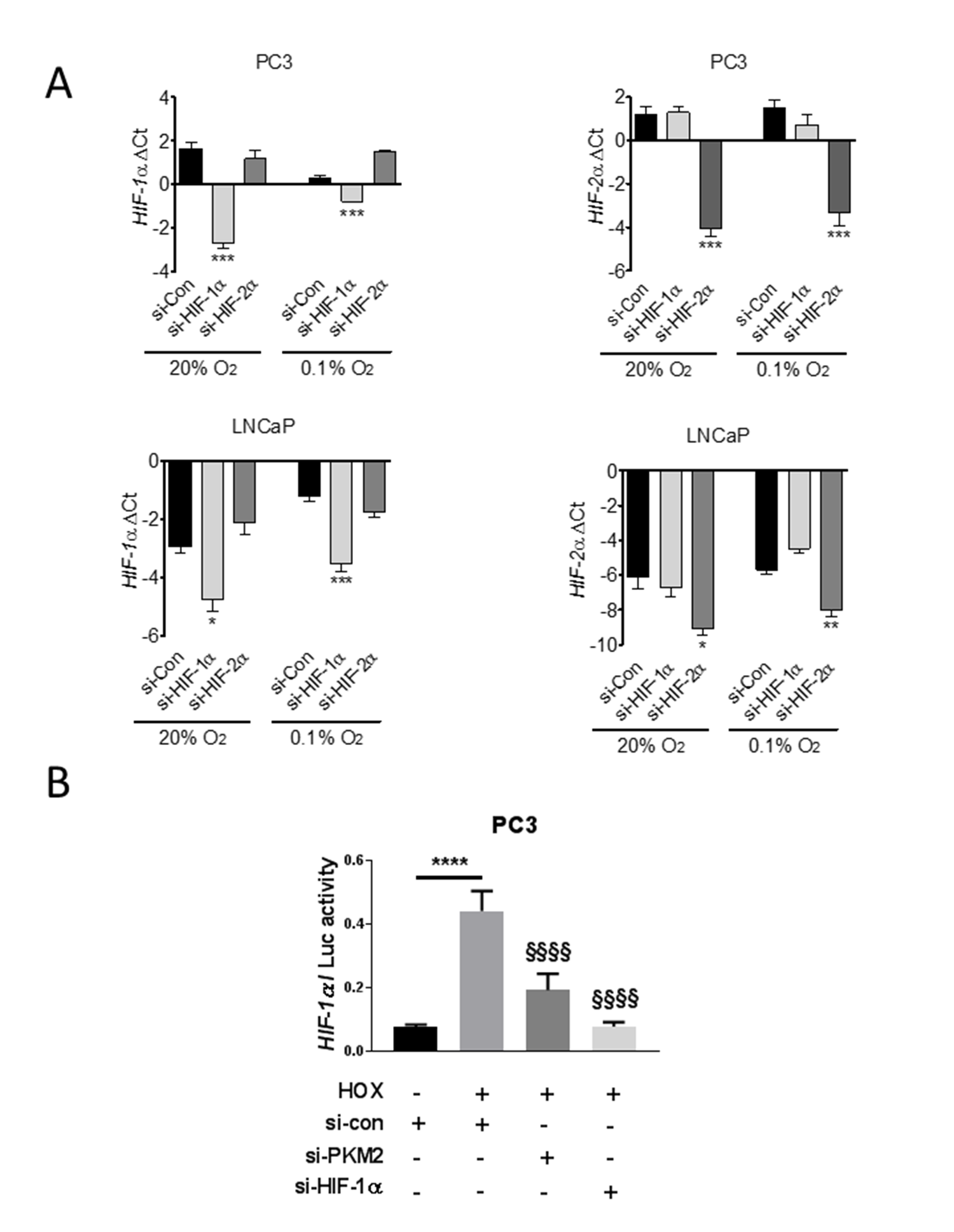

Supplement: S1 Fig — PC3 and LNCaP cells were transfected and cultured for 24 h in normoxia 20% O2 or hypoxia 0.1% O2. (A) HIF-1α and HIF-2α mRNA expression detection and quantification by qRT-PCR after treatment of cells with si-HIF-1α or si-HIF-2α (n = 4, Mean ± SEM, * P < 0.05, ** P < 0.01, *** P < 0.01 vs si-con Nox or Hox, ANOVA). (B) HRE reporter gene assay of transfected cells with si-PKM2 and si-HIF-1α (n = 6, Mean ± SEM, **** P < 0.0001, §§§§ P < 0.0001 vs si-con Hox). (TIF) [file pone.0203745.s001.tif]

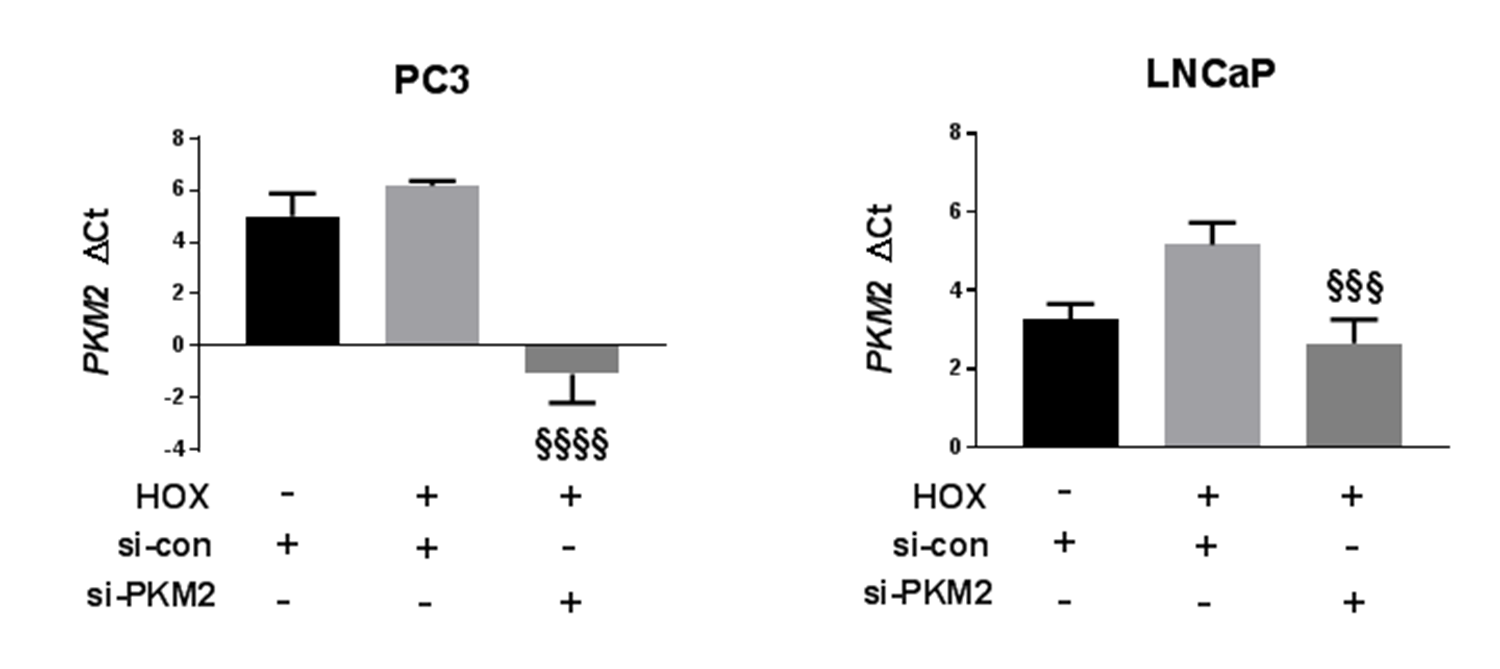

Supplement: S2 Fig — PC3 and LNCaP cells were transfected and cultured for 24 hr in normoxia 20% O2 or hypoxia 0.1% O2. PKM2 mRNA expression detection and quantification by qRT-PCR after treatment of cells with si-PKM2 (n = 4, Mean ± SEM, *** P < 0.001, **** P < 0.0001 vs si-con Hox). (TIF) [file pone.0203745.s002.tif]

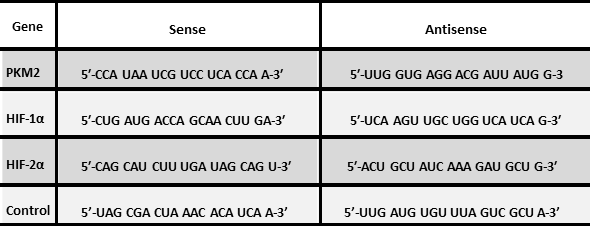

Supplement: S1 Table — (TIF) [file pone.0203745.s003.tif]

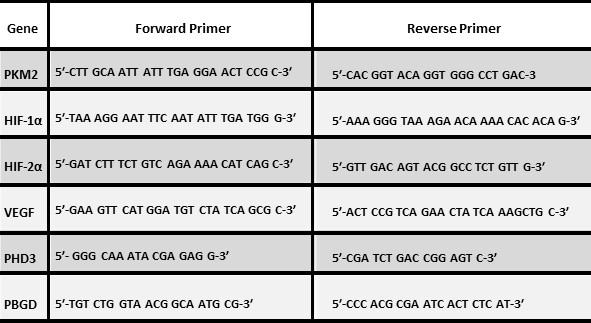

Supplement: S2 Table — (TIF) [file pone.0203745.s004.tif]
